# Overall Survival Improvement in Patients with Epidermal Growth Factor Receptor-Mutated Non-Small Cell Lung Cancer and Bone Metastasis Treated with Denosumab

**DOI:** 10.3390/cancers14143470

**Published:** 2022-07-17

**Authors:** How-Wen Ko, Chi-Tsun Chiu, Chih-Liang Wang, Tsung-Ying Yang, Chien-Ying Liu, Chih-Teng Yu, Li-Chuan Tseng, Chih-Hsi Scott Kuo, Chin-Chou Wang, Muh-Hwa Yang, Cheng-Ta Yang

**Affiliations:** 1 Division of Thoracic Oncology, Department of Thoracic Medicine, Linkou Chang Gung Memorial Hospital, College of Medicine, Chang Gung University, Taoyuan 333, Taiwan; howwenko@gmail.com (H.-W.K.); wang@cgmh.org.tw (C.-L.W.); cyliu01@cgmh.org.tw (C.-Y.L.); yuokao@ms19.hinet.net (C.-T.Y.); yang1946@cgmh.org.tw (C.-T.Y.); 2 Institute of European and American Studies, Academia Sinica, Taipei 115, Taiwan; ctchiu@gate.sinica.edu.tw; 3 Division of Chest Medicine, Department of Internal Medicine, Taichung Veterans General Hospital, Taichung 407, Taiwan; jonyin@gmail.com; 4 Department of Life Sciences, National Chung Hsing University, Taichung 402, Taiwan; 5 Department of Oncology Case Management, Linkou Chang Gung Memorial Hospital, Taoyuan 333, Taiwan; chientseng@cgmh.org.tw; 6 Division of Pulmonary & Critical Care Medicine, Department of Internal Medicine, Kaohsiung Chang Gung Memorial Hospital, Kaohsiung 833, Taiwan; ccwang5202@yahoo.com.tw; 7 Institute of Clinical Medicine, National Yang Ming Chiao Tung University, Taipei 112, Taiwan; mhyang2@vghtpe.gov.tw; 8 Division of Medical Oncology, Taipei Veterans General Hospital, Taipei 112, Taiwan; 9 Department of Thoracic Medicine, Taoyuan Chang Gung Memorial Hospital, Taoyuan 333, Taiwan; 10 Department of Respiratory Therapy, College of Medicine, Chang Gung University, Taoyuan 333, Taiwan

**Keywords:** denosumab, *EGFR*, NSCLC, bone metastasis, SRE, overall survival

## Abstract

**Simple Summary:**

Denosumab, a bone-modifying agent, has been approved for the prevention of or reduction in skeletal-related events (SREs) in non-small cell lung cancer (NSCLC) patients with bone metastasis. However, the effect of denosumab on survival of epidermal growth factor receptor (*EGFR*)-mutated NSCLC patients with bone metastasis has been insufficiently investigated. The present study showed that denosumab treatment was significantly correlated with improved overall survival (OS) in *EGFR*-mutated NSCLC patients with bone metastasis. In subgroup analyses, denosumab adjuvant therapy prolonged SRE-free survival (SRE-FS) in patients without initial SREs and was correlated with a better OS in patients with initial or pre-existing SREs. This study provided novel evidence of the survival benefit of denosumab for *EGFR*-mutated NSCLC patients with bone metastasis.

**Abstract:**

The impact of an initial skeletal-related event (SRE) and denosumab adjuvant treatment on the survival outcome of epidermal growth factor receptor (*EGFR*)-mutated non-small cell lung cancer (NSCLC) patients with bone metastasis remains unclear. This retrospective study included 400 metastatic *EGFR*-mutated NSCLC patients. Among 190 bone metastasis patients, 61 had initial SREs and 73 received denosumab. We analyzed patient characteristics, SRE-free survival (SRE-FS), and overall survival (OS). In metastatic *EGFR*-mutated NSCLC, bone metastasis was associated with a poorer OS (21.7 vs. 33.0 months; *p* < 0.001). Bone metastasis patients with initial SREs at diagnosis had an even shorter OS, compared with those without initial SRE (15.4 vs. 23.6 months; *p* = 0.026). Denosumab reduced SRE incidence (hazard ratio (HR) 0.57 (95% confidence interval (CI) 0.34–0.94; *p* = 0.027) and was associated with improved OS (26.6 vs. 20.1 months; *p* = 0.015). A multivariate analysis demonstrated that denosumab treatment was correlated with a lower incidence of SRE (HR 0.61 (95% CI 0.37–0.98); *p* = 0.042) and better OS (HR 0.60 (95% CI 0.41–0.88); *p* = 0.008). In subgroup analyses, denosumab prolonged SRE-FS (HR 0.36 (95% CI 0.19–0.79); *p* = 0.009) in patients without initial SREs and was related to a better OS (25.3 vs. 12.9 months; *p* = 0.016) in patients with initial or pre-existing SREs. Osteonecrosis of the jaw was diagnosed in two patients (2.74%) receiving denosumab. Our study confirmed the association between initial SREs and a worse outcome and provided novel evidence of the survival benefit of denosumab for *EGFR*-mutated NSCLC patients with bone metastasis.

## 1. Introduction 

Advanced non-small cell lung cancer (NSCLC) has a poor prognosis and has a high incidence of bone metastasis. Approximately one-third of stage IV NSCLC patients present with bone metastasis at diagnosis [1,2], with a median overall survival (OS) of less than 9 months [1,2,3,4]. About 50% of Asian and 11–16% of non-Asian NSCLC patients harbor epidermal growth factor receptor (*EGFR*) mutations [5]. Studies have shown that patients carrying *EGFR* mutations have a higher incidence of distant metastasis and are prone to the development of bone metastasis [2,6]. Randomized clinical trials have shown that front-line treatment with first-, second-, and third-generation *EGFR*-tyrosine kinase inhibitors (TKIs) greatly improved the survival outcomes of advanced *EGFR*-mutated NSCLC patients [5]. However, investigations about the prognosis of *EGFR*-mutated NSCLC patients with bone metastasis are limited [7].

Patients with bone metastasis frequently experience pain and skeletal-related events (SREs), including pathologic fracture, spinal cord compression, hypercalcemia, and the need for bone surgery or radiation therapy, which cause significant morbidity [8,9,10]. Spine metastasis can cause intractable pain, spinal instability, and more serious SREs, thereby decreasing patients’ quality of life. One of the most devastating SREs is spinal cord compression, which represents an oncologic emergency [11]. Once it is diagnosed, steroid therapy should be administered immediately, followed by surgical evaluation and adjuvant radiotherapy. More than 40% of NSCLC patients with bone metastasis develop SREs [3,9,12], with a median time to first event of less than 6 months [9,12,13]. Retrospective surveys have reported that NSCLC patients with SREs tend to have shorter OS than those without SRE [13,14]. It is worth noting that a subgroup of these patients had SRE at the time of initial NSCLC diagnosis (described as initial SRE) [15], and possibly leading to worse outcomes. However, few studies have investigated this subgroup population. Moreover, the prevalence of SREs is likely to increase in *EGFR*-mutated patients with the increase in their survival.

The current strategy to manage bone metastasis is to prevent or delay the occurrence of SREs. The bisphosphonates, zoledronic acid and denosumab, are two major systemic bone-modifying agents (BMAs) that block the activity of the osteoclasts [9,10]. A phase III clinical trial validated the efficacy of zoledronic acid in delaying and reducing SREs without achieving a survival benefit in lung cancer patients [9]. A subsequent study demonstrated a superior tendency of denosumab over zoledronic acid in terms of therapeutic effectiveness [10]. Denosumab is a fully human monoclonal antibody that inhibits the receptor activator of nuclear factor kappa B ligand (RANKL), which is an essential mediator of bone resorption. Inhibition of RANKL prevents the formation, function, and survival of osteoclasts [16]. In an NSCLC mouse model of bone metastasis, suppression of RANKL reduced the skeletal tumor burden and prolonged its survival [17]. An exploratory subgroup analysis revealed that lung cancer patients treated with denosumab had a better OS than those treated with zoledronic acid [3]. The impact of denosumab on the survival of NSCLC patients with *EGFR* mutations remains elusive.

In the present study, we aimed to investigate whether denosumab affects the outcome of *EGFR*-mutated NSCLC patients with bone metastasis in a real-world cohort. In addition to the influence of SREs at initial diagnosis on the OS of patients with bone metastasis, we also examined the effect of denosumab on the prognosis of patients with and without initial or preexisting SREs.

## 2. Methods

### 2.1. Study Design and Patient Population

**Figure 1:**
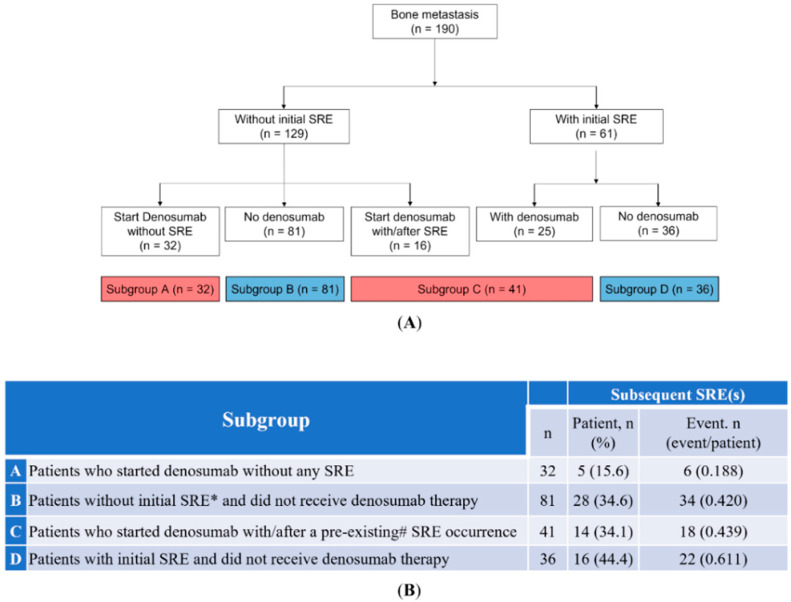
(**A**) Subgroups of *EGFR*-mutated NSCLC patients with bone metastasis, and (**B**) subsequent SRE(s) occurrence in different subgroups of *EGFR*-mutated NSCLC patients with bone metastasis. * Initial SRE: SRE occurring at the time of initial diagnosis of NSCLC concomitant with bone metastasis. # Pre-existing SRE: the SRE leading to initiation of denosumab. *EGFR*: epidermal growth factor receptor; NSCLC: non-small cell lung cancer; SRE: skeletal-related event.

This was a retrospective cohort study which used data from the Chang Gung Research Database, a multi-institutional electronic medical records collection in Taiwan [18]. Patients who were treated at Chang Gung Memorial Hospital between January 2016 and January 2018 were retrospectively screened. The inclusion criteria were: (1) newly diagnosed or recurrent metastatic NSCLC; (2) positive *EGFR* mutation; and (3) receiving Taiwan’s National Health Insurance reimbursed first-line treatment with gefitinib, erlotinib, or afatinib. All patients underwent a staging assessment at diagnosis, including a chest tomography (CT) scan, positron emission tomography (PET) scan, bone scan, and/or brain imaging. The exclusion criteria were: (1) treatment duration of less than 1 month; and (2) receiving BMAs other than denosumab. Patients with bone metastatic lesion by contiguity were considered as having no bone metastasis. Clinical data were recorded, including age, sex, smoking status, Eastern Cooperative Oncology Group (ECOG) performance status, histology, disease stage (American Joint Committee on Cancer, 8th edition), *EGFR* mutation types, first-line TKI treatment, initial metastasis before the administration of *EGFR*-TKI, and the number of bone metastatic site(s). The number and types of SRE were collected. A subsequent SRE was defined as an event occurring more than 1 month after the previous SRE and was not related to the previous SRE. The use of denosumab treatment, number of cycles, treatment duration, and incidence of adverse effects were also reviewed. For subgroup analysis, all enrolled participants were divided into the following four subgroups: (A) patients who started denosumab without any SRE, (B) patients without SRE at the initial diagnosis who did not receive denosumab therapy, (C) patients who started denosumab with/after a pre-existing occurrence of SRE; and (D) patients with SREs at the initial diagnosis who did not receive denosumab therapy (Figure 1). This study was approved by the Institutional Review Board of the Chang Gung Medical Foundation (No. 202101164B0) and was conducted in accordance with the Good Clinical Practice guidelines and the Declaration of Helsinki. All participant data were anonymized and the need for written informed consent was waived.

### 2.2. Statistical Analysis

All the data of the enrolled patients were included in the analysis. The data cut-off for the final analysis was 1 June 2021. Categorical variables were compared using Fisher’s exact test. Continuous variables were evaluated using Student’s *t*-test. Survival curves were calculated using the Kaplan–Meier method and compared using the log-rank test. A Cox proportional hazards regression model was employed to calculate the hazard ratios (HRs) and 95% confidence intervals (CIs) for univariate and multivariate analyses to identify the determinants of OS in patients with bone metastasis. To assess the risk factors for the occurrence of SREs, a multiple-event analysis was performed using the Andersen and Gill model [10]. SRE-free survival (SRE-FS) was also analyzed using the Kaplan–Meier method and compared using the log-rank test. In patients who started denosumab treatment without initial or preexisting SRE, the SRE-FS was defined as the time between the start date of first-line TKI treatment and the date of the first SRE occurrence, or was censored at the last date of follow-up. The above definition was the same in patients who had no SRE at the initial diagnosis and did not receive denosumab treatment. For patients who started denosumab after or at the time of the SRE occurrence (described as pre-existing SRE), SRE-FS was calculated from the date of the occurrence of SRE consequently requiring denosumab treatment initiation to the date of the next SRE recurrence or was censored at the last date of follow-up. In patients who had SREs at the initial diagnosis and did not receive denosumab treatment, it was estimated from the date of the first SRE occurrence to the date of the next SRE recurrence or was censored at the last date of follow-up. A two-sided *p* value of less than 0.05 was considered statistically significant. Statistical analyses were performed using GraphPad Prism (version 5.02, GraphPad Software, La Jolla, CA, USA) and R software (version 4.1.2).

## 3. Results

### 3.1. Patient Characteristics

A total of 400 metastatic NSCLC patients harboring *EGFR* mutations and receiving first-line TKI treatment were enrolled. Among them, 386 patients were newly diagnosed and 14 patients had disease recurrence from earlier stages. Overall, 25 patients were excluded as 23 patients had a treatment duration of less than 1 month or were lost to follow-up, and two patients received BMAs other than denosumab (Figure S1). Three patients who had bone metastatic involvement by contiguity were considered as having no bone metastasis. Baseline characteristics are summarized in Table 1.

**Figure 2:**
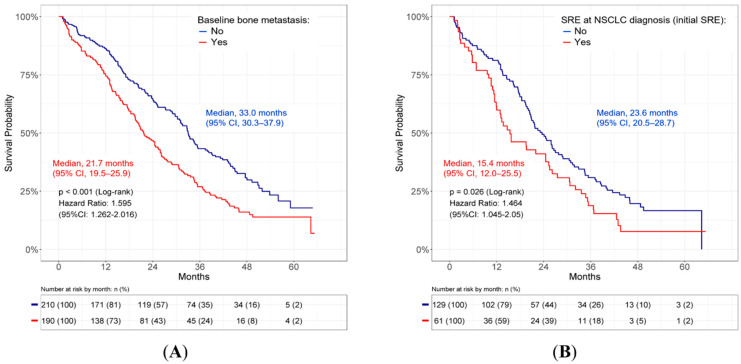
OS in (**A**) metastatic *EGFR*-mutated NSCLC patients with or without baseline bone metastasis and (**B**) bone metastatic patients with or without SRE at NSCLC diagnosis (initial SRE). CI, confidence interval; *EGFR*: epidermal growth factor receptor; NSCLC: non-small cell lung cancer, OS: overall survival; SRE: skeletal-related event.

The bone was the most common site of extrapulmonary metastases in patients with metastatic, *EGFR*-mutated NSCLC. One hundred and ninety (47.5%) patients had bone metastasis, and these patients were associated with younger age, poorer ECOG performance status, more advanced stage of the disease, and more liver and adrenal/renal metastases. Compared to those without bone involvement, patients with bone metastasis had significantly shorter OS (median, 21.7 months (95% confidence interval (CI): 19.5–25.9) vs. 33.0 months (95% CI: 30.3–37.9); *p* < 0.001; Figure 2A). Multivariate Cox regression analyses further identified bone metastasis as a determinant of poorer OS (HR = 1.37, 95% CI 1.07–1.76, *p* = 0.013; Table S1).

Among patients with bone metastasis, 110 (57.9%) experienced SREs, and 61 patients (32.1%) had SREs at the time of the initial NSCLC diagnosis. Patients with initial SREs had an even shorter OS, compared with those without pre-existing SRE (median, 15.4 months (95% CI: 12.0–25.5) vs. 23.6 months (95% CI: 20.5–28.7); *p* = 0.026; Figure 2B).

### 3.2. The Effect of Denosumab

**Figure 3:**
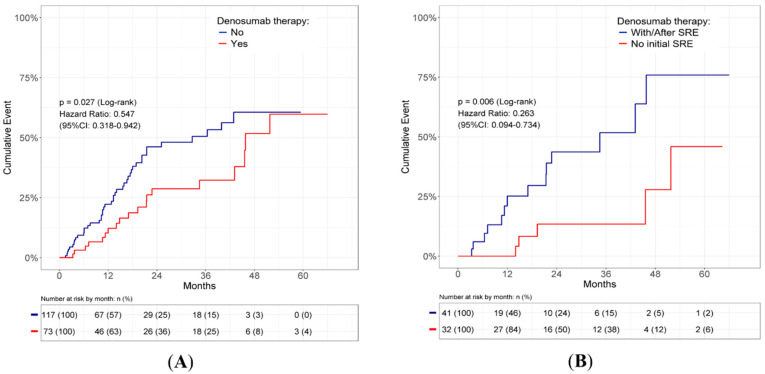
Denosumab effect on subsequent SREs occurrence. (**A**) Cumulative incidence of subsequent SREs in *EGFR*-mutated NSCLC patients with bone metastasis with or without denosumab therapy. (**B**) Cumulative incidence of subsequent SREs in *EGFR*-mutated NSCLC patients with bone metastasis who started denosumab therapy without initial SREs or with/after SREs. CI: confidence interval; *EGFR*: epidermal growth factor receptor; NSCLC: non-small cell lung cancer; SRE: skeletal-related event.

Of the patients with bone metastasis, 73 patients (38.4%) received denosumab treatment. The demographic data of the patients treated with and without denosumab are shown in Table 1. There were no significant differences in any of the variables between the two groups. The use of denosumab significantly reduced the occurrence of subsequent SREs in patients with bone metastasis compared to those without denosumab treatment (HR = 0.55, 95% CI 0.32–0.94, *p* = 0.027; Figure 3A). Overall, 32 (43.8%) of the 73 patients began receiving denosumab without any pre-existing SREs. The remaining 41 patients (56.2%) initiated denosumab treatment at the time of or after the occurrence of SREs. The incidence of subsequent SREs was significantly lower in patients without any pre-existing SREs than in those with pre-existing SREs (HR = 0.26, 95% CI 0.09–0.73, *p* = 0.006; Figure 3B). A multiple-event analysis using the Andersen and Gill model was employed to determine the risk factors that affect the occurrence of subsequent SREs. Pathologic fractures (HR = 5.45, 95% CI 3.29–9.03, *p* < 0.001) and bone radiation therapy (HR = 3.43, 95% CI 1.86–6.32, *p* < 0.001) were correlated with a higher incidence of subsequent SREs. In contrast, denosumab treatment (HR = 0.53, 95% CI 0.31–0.90, *p* = 0.019) was the only factor associated with a lower incidence of SREs (Table 2).

**Figure 4:**
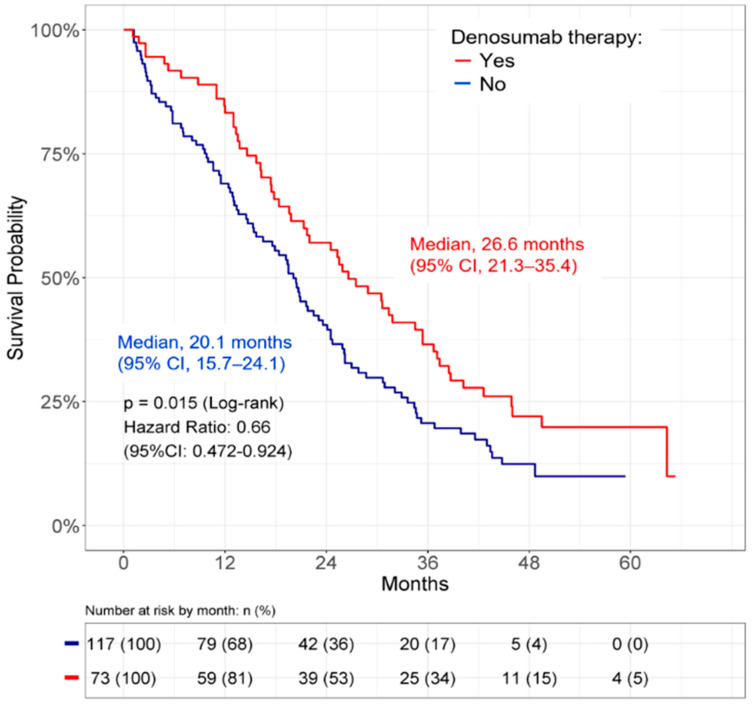
Denosumab effect on OS in *EGFR*-mutated NSCLC patients with bone metastasis with or without denosumab therapy. CI: confidence interval; *EGFR*: epidermal growth factor receptor; NSCLC: non-small cell lung cancer; OS: overall survival.

Upon investigating the outcomes, the use of denosumab was found to be correlated with a longer OS compared with the group not receiving denosumab (median, 26.6 months (95% CI: 21.3–35.4) vs. 20.1 months (95% CI: 15.7–24.1); *p* = 0.015; Figure 4). Univariate and multivariate analyses were performed to identify the independent factors that affected OS. An ECOG PS 2–4 (HR = 1.93, 95% CI 1.26–2.96, *p* = 0.002) and adrenal/renal metastases (HR = 2.91, 95% CI 1.83–4.65, *p* < 0.001) were associated with poorer OS. In contrast, afatinib treatment (HR = 0.57, 95% CI 0.39–0.83, *p* = 0.004) and the use of denosumab (HR = 0.59, 95% CI 0.41–0.87, *p* = 0.007) were predictive of longer OS (Table 3).

As the multivariate analysis showed that both afatinib and denosumab treatment reduced the relative risk, we further compared survival between afatinib/denosumab combination therapy and afatinib monotherapy. No denosumab monotherapy group was enrolled in the analysis because no patient was administered denosumab without any anticancer therapy. Of the 120 patients receiving first-line afatinib, 32 were treated with denosumab during the period of first-line afatinb treatment and 71 were not. The remaining 17 who received denosumab after discontinuation of first-line afatinib therapy were not included in the comparison. The results demonstrated that there was no OS difference between the afatinib/denosumab and afatinib alone groups (median, 25.9 months (95% CI: 18.4–42.6) vs. 26.2 months (95% CI: 20.8–34.4); *p* = 0.843).

### 3.3. Subgroup Analysis

**Figure 5:**
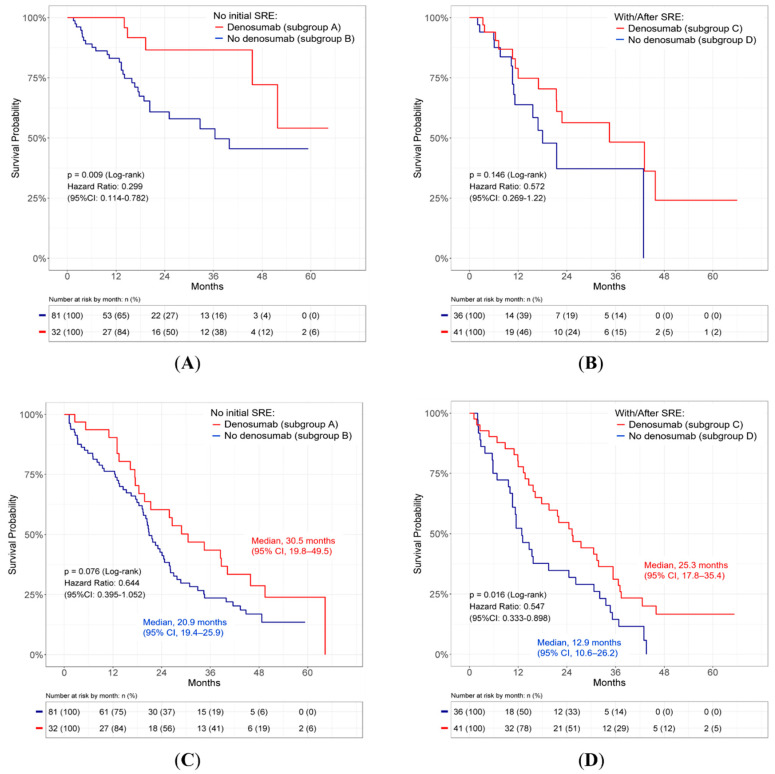
Subgroup analyses of denosumab’s effect on (**A**) SRE-FS in patients without initial SRE who did or did not receive denosumab therapy, (**B**) SRE-FS in patients with pre-existing SREs who received denosumab therapy or in patients with initial SREs who did not receive denosumab therapy, (**C**) OS in patients without initial SREs who did or did not receive denosumab therapy, and (**D**) OS in patients with pre-existing SREs who received denosumab therapy or in patients with initial SREs who did not receive denosumab therapy. CI: confidence interval; *EGFR*: epidermal growth factor receptor; NSCLC: non-small cell lung cancer; OS: overall survival; SRE-FS: skeletal-related event-free survival.

To understand the impact of denosumab treatment and the effect of initial or pre-existing SREs on the SRE-FS and OS of these patients, 190 patients were divided into four subgroups (Figure 1A). The number of patients with subsequent occurrence of SREs and the total SREs in each subgroup are listed in Figure 1B. SRE-FS was first evaluated. In the group of patients without SREs at initial diagnosis, the patients receiving denosumab treatment had a significantly longer SRE-FS than patients without treatment (HR = 0.30, 95% CI 0.11–0.78; *p* = 0.009; Figure 5A). In patients who had pre-existing SREs or SREs at initial diagnosis, no significant change was observed in the occurrence of subsequent SREs between patients receiving denosumab treatment and the other group not receiving denosumab treatment (HR = 0.57, 95% CI 0.27–1.22, *p* = 0.146; Figure 5B). OS was then assessed. In patients who had no SREs at the initial diagnosis, denosumab treatment tended to prolong OS without statistical significance compared to the other group not receiving treatment (median, 30.5 months [95% CI: 19.8–49.5] vs. 20.9 months [95% CI: 19.4–25.9]; *p* = 0.076; Figure 5C). In patients with pre-existing SREs or SREs at the initial diagnosis, denosumab treatment was associated with a significantly longer OS than those without treatment (median, 25.3 months (95% CI: 17.8–35.4) vs. 12.9 months (95% CI: 10.6–26.2); *p* = 0.016; Figure 5D).

### 3.4. Adverse Events

In the 73 patients receiving denosumab, the median number of cycles of treatment was six (range, 1–57). One patient (1.37%) complained of gomphosis and two patients (2.74%) experienced hypocalcemia. Medication-related osteonecrosis of the jaw was diagnosed in two patients (2.74%). Thus, denosumab was discontinued in patients with adverse events.

## 4. Discussion

To our knowledge, this was the first study to show that denosumab treatment was significantly associated with longer OS in *EGFR*-mutated NSCLC patients with bone metastasis. In addition to the observation of the association between initial SREs and a worse outcome, our findings demonstrate that denosumab adjuvant therapy was correlated with a longer SRE-FS in patients without initial SREs and an improved OS in patients with initial or pre-existing SREs.

Studies have reported that patients harboring *EGFR* mutations are susceptible to developing bone metastasis [2,6]. The prognosis of NSCLC patients with bone metastases is relatively poor [1,2,3,4]. In this investigation, the skeletal system was found to be the most common extrapulmonary metastatic site (Table 1). Compared to other stage IV participants, bone metastasis was correlated with younger age, poorer ECOG performance status, more advanced stage (Table 1), shorter OS (Figure 2A), and was an independent factor for poorer outcome (Table S1). This prompted us to further investigate this population.

The presence of SREs is reportedly associated with poor survival [12,13]. Among patients with SREs, a proportion had SREs at the time of initial diagnosis of NSCLC concomitant with bone metastasis [15]. However, little is known about the prognosis of these patients. In our study, SREs occurred in 110 (57.9%) of 190 patients, and SREs at the initial diagnosis were found in 61 (32.1%) patients (Table 1). Patients with SREs at the initial diagnosis had a worse OS (Figure 2B). This implies that SREs may further deteriorate the outcome of patients with bone metastasis.

Bone-modifying therapies have been recommended for NSCLC patients with bone metastasis [19,20]. Taiwan National Health Insurance introduced a reimbursement programme for the use of denosumab on 1 December 2015. Subsequently, denosumab has become the most commonly used BMA in Taiwan. Despite this, not all NSCLC physicians prescribe BMAs to manage bone diseases or SREs [21,22,23,24,25]. In addition, physicians and/or patients often decide to initiate bone treatment upon the occurrence of SREs or later in clinical practice. The real-world situation allowed us to assess the influence of SREs at the initial diagnosis or pre-existing SREs and denosumab treatment on SRE-FS and OS in these patients. Therefore, we divided the population into four subgroups (Figure 1).

The major therapeutic effect of denosumab is to prevent or delay the occurrence of SREs, which was confirmed in the present study (Figure 3 and Table 2). It remains unclear whether the effect is similar between NSCLC patients with and without pre-existing SREs or SREs at the initial diagnosis. In the present study, we found that denosumab significantly prolonged SRE-FS in patients without SREs at the initial diagnosis (Figure 5A); additionally, it tended to delay the occurrence of SREs in patients who had pre-existing SREs or SREs at the initial diagnosis without statistical significance (Figure 5B). Likewise, we observed that denosumab significantly diminished the incidence of SREs in patients without initial SREs, compared to the incidence of SREs recurrence in patients with pre-existing SREs or SREs at initial diagnosis (Figure 3B). These results suggest that denosumab may be more effective in patients without SREs at initial diagnosis than in those with pre-existing SREs or SREs at initial diagnosis. The results also imply that the initiation time of denosumab should be earlier to prevent the occurrence of SREs in *EGFR*-mutated NSCLC patients with bone metastasis.

A small number of clinical studies have reported that denosumab is beneficial for the improvement of OS in NSCLC patients with bone metastasis [3,21,26,27]. The potential factors contributing to the survival benefit of denosumab remain unclear. A fundamental rationale based on pre-clinical research is that RANKL inhibition may have direct and indirect anti-tumor effects in addition to bone remodeling [16]. Another hypothesis is that the therapeutic effect of denosumab involves SREs reduction, which may prevent an exacerbation of the performance status and prolong survival. However, two recent analyses failed to show the advantage of denosumab in NSCLC patients receiving chemotherapies and immunotherapies [4,22]. This evidence prompted us to examine whether denosumab has beneficial effects in certain NSCLC subgroups. The results of our investigation further identified the beneficial effects of denosumab in NSCLC patients with *EGFR* mutations (Figure 4 and Table 3). In the comparison of patient groups, we did not observe statistical OS differences between afatinib/denosumab combination therapy and afatinib monotherapy groups. Since SREs at the initial diagnosis were found to be associated with shorter OS (Figure 2B), we next explored the impact of initial or pre-existing SREs on the survival improvement by denosumab. Intriguingly, we found that denosumab was significantly associated with a longer OS in patients with pre-existing SREs or SREs at the initial diagnosis (Figure 5D). In patients who had no SREs at the initial diagnosis, denosumab tended to enhance survival without statistical significance (Figure 5C). These data suggest that the correlation between denosumab treatment and OS improvement in the *EGFR*-mutated population is possibly attributed to the reduction in SREs and prevention of exacerbations of performance status. These results further suggest the negative influence of pre-existing SREs or SREs at initial diagnosis on the OS of NSCLC patients with bone metastasis. Recently, Chiu et al. [23] reviewed 77 patients with *EGFR*-mutated bone metastasis. In their analysis, the median OS was better in the denosumab group compared to that in the no denosumab groups; however, no statistically significant difference was observed (29.5 vs. 26.9 months, *p* = 0.967). It is not clear whether the statistically insignificant result is due to an insufficient follow-up time, inadequate number of subjects, or other factors.

Although bone-modifying therapies have been approved for NSCLC patients with bone metastasis, only around half of NSCLC patients with bone metastasis received BMAs to manage bone diseases or SREs [21,22]. In Taiwan, a recent national-based survey investigated 44,800 lung cancer patients with bone metastasis and reported that BMAs had been prescribed in only 28.4% patients [25]. In the present study, seventy three (38.4%) patients had denosumab in addition to two receiving other BMAs and 56.1% of seventy three patients started denosumab treatment with or after SRE occurrence (Table 1 and Figure S1). Uncertain survival benefits and adverse effects such as jaw osteonecrosis may be part of the reasons causing the suboptimal treatment for lung cancer patients with bone metastasis. Our investigation provided novel evidence and clinical-based support for the use of denosumab in NSCLC patients with bone metastasis and *EGFR* mutation.

This study has several limitations. The first and major limitation is its retrospective nature. In real-world situations, the starting time for denosumab varies. It is challenging to assess the beneficial effect of denosumab on progression-free survival upon administrating *EGFR*-TKI treatment. Therefore, we explored OS as the primary indicator and calculated treatment-related SRE-FS to examine the effect of denosumab on SREs. Moreover, the retrospective nature prevented us from observing the actual denosumab-related SRE-FS. Prospective randomized control trials are required to validate our findings. The second limitation is the small number of cases. Denosumab has been reimbursed by Taiwan NHI since December 2015; accordingly, more patients received denosumab after 2016. Since recent studies [4,22,24] failed to demonstrate the OS advantage of denosumab, we supposed that a sufficiently long follow-up time is needed to evaluate its benefit. In the current study, the minimum follow-up duration was 40 months. Consequently, only a limited number of patients could be included in this study. The third limitation is that some variables were not collected, such as alkaline phosphatase levels, lactate dehydrogenase levels, pain assessment, characteristics of bone metastasis and other treatments such as anti-VEGF/VEGFR treatment. These variables may affect OS and the SRE-FS.

## 5. Conclusions

In summary, this study demonstrated an association between bone metastasis and poor survival outcomes in *EGFR*-mutated NSCLC patients with bone metastasis. Denosumab treatment was an independent prognostic factor for improved OS in these patients. The addition of denosumab was significantly correlated with prolonged SRE-FS in patients without initial SREs and extended OS in patients with initial or pre-existing SREs. Our study provided novel evidence of the survival benefit of denosumab for *EGFR*-mutated NSCLC patients with bone metastasis. Larger prospective clinical studies are required to validate our findings.

## Figures and Tables

**Table 1:** Baseline Characteristics of all metastatic *EGFR*-mutant NSCLC patients.

Characteristic, N (%)	Without Bone Metastasis N = 210	With Bone Metastasis, N = 190			P1
		With Denosumab	Without Denosumab	P2	
Age				0.072	0.005
≥65	126 (60.0)	27 (37.0)	60 (51.3)		
<65	84 (40.0)	46 (63.0)	57 (48.7)		
Sex				0.217	0.125
Male	92 (43.8)	22 (30.1)	46 (39.3)		
Female	118 (56.2)	51 (69.9)	71 (60.7)		
ECOG PS				0.466	0.022
0~1	184 (87.6)	60 (82.2)	90 (76.9)		
2~4	26 (12.4)	13 (17.8)	27 (23.1)		
Smoking status				0.414	0.078
Never	162 (77.1)	64 (87.7)	96 (82.1)		
Current/ex-smoker	48 (22.9)	9 (12.3)	21 (17.9)		
Histology				1.000	0.481
Adenocarcinoma	202 (96.2)	69 (94.5)	110 (94.0)		
Non-adenocarcinoma	8 (3.8)	4 (5.5)	7 (6.0)		
Stage				0.786	<0.001
M1a	114 (54.3)	–	–		
M1b	39 (18.6)	5 (6.8)	10 (8.5)		
M1c	57 (27.1)	68 (93.2)	107 (91.5)		
Metastasis					
with lung/pleura	164 (78.1)	49 (68.1)	83 (70.9)	0.628	0.053
with bone	–	73 (100.0)	117 (100.0)	–	
with brain with brain metastasectomy	68 (32.4) 8 (3.8)	27 (37.5) 2 (2.7)	45 (38.5) 1 (0.9)	0.879 0.560	0.251 0.227
with liver	17 (8.1)	14 (19.4)	19 (16.2)	0.694	0.006
with adrenal/renal	13 (6.2)	10 (13.9)	20 (17.1)	0.683	0.002
with abdominal LNs	15 (7.1)	8 (11.1)	8 (6.8)	0.421	0.709
*EGFR* mutation				1.000	0.318
Exon 19 deletion	93 (44.3)	31 (42.5)	50 (42.7)		
L858R and uncommon	117 (55.7)	42 (57.5)	67 (57.3)		
First-line *EGFR*-TKI				0.440	0.541
Gefitinib/Erlotinib	84 (40.0)	24 (32.9)	46 (39.3)		
Afatinib	126 (60.0)	49 (67.1)	71 (60.7)		
No. of bone metastatic site				0.078	–
Single	–	8 (11.0)	25 (21.4)		
2 or more	–	65 (89.0)	92 (78.6)		
Patients with SRE				0.292	–
No	–	27 (37.0)	53 (45.3)		
Yes	–	46 (63.0)	64 (54.7)		
Initial SRE *				0.635	–
No	–	48 (65.8)	81 (69.2)		
Yes	–	25 (34.2)	36 (30.8)		
SRE type					
Pathologic fracture	–	23 (31.5)	41 (26.9)	0.640	–
Spinal cord compression	–	10 (13.7)	11 (11.7)	0.746	–
Hypercalcemia	–	1 (1.4)	3 (1.2)	1.000	–
Bone surgery	–	12 (16.4)	21 (14.0)	0.846	–
Bone radiation therapy	–	39 (53.4)	45 (45.7)	0.051	–
Denosumab treatment					–
Start without any SRE	–	32 (43.8)	–		
Start with/after SRE	–	41 (56.2)	–		

P1: comparison between *EGFR*-mutated NSCLC patients with and without bone metastasis. P2: comparison between bone metastatic patients with and without denosumab treatment. ECOG PS: Eastern Cooperative Oncology Group performance status; *EGFR*: epidermal growth factor receptor; LNs: lymph nodes; NSCLC: non-small cell lung cancer; TKI: tyrosine kinase inhibitor; SRE: skeletal-related event. * SRE at the time of initial NSCLC diagnosis.

**Table 2:** Multiple-event analysis * of subsequent SRE occurrence of *EGFR*-mutated NSCLC patients with bone metastasis.

Variable	Univariate Analysis			Multivariate Analysis		
	HR	95% CI	*p* Value	HR	95% CI	*p* Value
Age						
≥65	1.226	0.751–2.002	0.416	–	–	–
Sex						
Female	1.298	0.790–2.131	0.304	–	–	–
ECOG PS						
2~4	1.449	0.830–2.530	0.192	–	–	–
Smoking status						
Current/ex-smoker	1.597	0.887–2.878	0.119	–	–	–
Histology						
Adenocarcinoma	0.672	0.237–1.910	0.456	–	–	–
Metastasis						
with lung/pleura/pericardia	0.693	0.420–1.145	0.152	–	–	–
with brain	1.001	0.627–1.598	0.995	–	–	–
with liver	0.574	0.260–1.266	0.169	–	–	–
with adrenal/renal	1.704	0.879–3.302	0.114	–	–	–
with abdominal LNs/spleen	0.693	0.219–2.193	0.532	–	–	–
*EGFR* mutation						
Exon 19 deletion	0.922	0.559–1.521	0.751	–	–	–
First-line *EGFR*-TKI						
Afatinib	0.656	0.396–1.088	0.103	–	–	–
Number of bone metastatic site						
2 or more	1.627	0.794–3.334	0.184	–	–	–
SRE at NSCLC diagnosis (initial SRE)						
Yes	2.193	1.354–3.550	0.001	0.987	0.567–1.718	0.963
SRE types						
Pathologic fracture	5.178	3.225–8.313	<0.001	5.450	3.289–9.029	<0.001
Spinal cord compression	1.902	1.046–3.459	0.035	1.056	0.571–1.953	0.863
Hypercalcemia	2.462	1.016–5.965	0.046	2.318	0.989–5.435	0.053
Bone surgery	2.831	1.708–4.693	<0.001	1.089	0.690–1.719	0.713
Bone radiation therapy	3.571	2.114–6.033	<0.001	3.425	1.857–6.318	<0.001
Denosumab therapy						
Yes	0.510	0.306–0.852	0.010	0.528	0.309–0.902	0.019

* using the Andersen and Gill model. CI: confidence interval; ECOG PS: Eastern Cooperative Oncology Group performance status; *EGFR*: epidermal growth factor receptor; HR: hazards ratio; LNs: lymph nodes; NSCLC: non-small cell lung cancer; TKI: tyrosine kinase inhibitor; SRE: skeletal-related event.

**Table 3:** Cox regression analysis of overall survival of *EGFR*-mutated NSCLC patients with bone metastasis.

Variable	Univariate Analysis			Multivariate Analysis		
	HR	95% CI	*p* Value	HR	95% CI	*p* Value
Age						
≥65	1.092	0.790–1.511	0.594	–	–	–
Sex						
Female	0.799	0.566–1.127	0.200	–	–	–
ECOG PS						
2~4	2.420	1.667–3.512	<0.001	1.932	1.261–2.959	0.002
Smoking status						
Current/ex-smoker	0.910	0.588–1.409	0.673	–	–	–
Histology						
Adenocarcinoma	0.638	0.336–1.213	0.171	–	–	–
Metastasis						
with lung/pleura/pericardia	1.315	0.924–1.871	0.129	–	–	–
with brain	1.038	0.743–1.449	0.827	–	–	–
with liver	1.396	0.924–2.110	0.113	–	–	–
with adrenal/renal	2.229	1.466–3.390	<0.001	2.914	1.825–4.654	<0.001
with abdominal LNs/spleen	1.279	0.723–2.263	0.397	–	–	–
*EGFR* mutation						
Exon 19 deletion	0.789	0.569–1.095	0.156	–	–	–
First-line *EGFR*-TKI						
Afatinib	0.562	0.404–0.781	0.001	0.567	0.387–0.832	0.004
Number of bone metastatic site						
2 or more	1.157	0.931–1.437	0.502	–	–	–
SRE at NSCLC diagnosis (initial SRE)						
Yes	1.464	1.045–2.050	0.027	1.659	0.971–2.834	0.064
SRE type						
Pathologic fracture	1.701	1.219–2.373	0.002	1.422	0.964–2.099	0.076
Spinal cord compression	1.165	0.718–1.892	0.536	–	–	–
Hypercalcemia	3.279	1.198–8.975	0.021	0.872	0.275–2.764	0.817
Bone surgery	1.158	0.774–1.731	0.475	–	–	–
Bone radiation therapy	0.937	0.679–1.293	0.692	–	–	–
Denosumab use						
Yes	0.660	0.472–0.924	0.016	0.594	0.408–0.865	0.007

CI, confidence interval; ECOG PS, Eastern Cooperative Oncology Group performance status; *EGFR*: epidermal growth factor receptor; HR: hazards ratio; LNs: lymph nodes; NSCLC: non-small cell lung cancer; TKI: tyrosine kinase inhibitor; SRE: skeletal-related event.

## Data Availability

The data presented in this study are available on request from the corresponding author.

## Supplementary Materials

The following supporting information can be downloaded at: https://www.mdpi.com/article/10.3390/cancers14143470/s1, Figure S1. Flowchart of patient enrollment in this study, Table S1. Cox regression analysis of OS of all metastatic *EGFR*-mutated NSCLC patients.
