# Supplementary material for: Overall Survival Improvement in Patients with Epidermal Growth Factor Receptor-Mutated Non-Small Cell Lung Cancer and Bone Metastasis Treated with Denosumab
Source: Cancers (Basel). 2022 Jul 17;14(14):3470. doi: 10.3390/cancers14143470 (PMC9316991; doi:10.3390/cancers14143470)
Supplement: Supplementary file 1 [file cancers-14-03470-s001.zip › cancers-1811781-supplementary.pdf]

cancers MDPI

Article

Overall Survival Improvement in Patients with Epidermal Growth Factor Receptor-Mutated Non-Small Cell Lung Cancer and Bone Metastasis Treated with Denosumab

How-Wen Ko, Chi-Tsun Chiu, Chih-Liang Wang, Tsung-Ying Yang, Chien-Ying Liu, Chih-Teng Yu, Li-Chuan Tseng, Chih-Hsi Kuo, Chin-Chou Wang, Muh-Hwa Yang and Cheng-Ta Yang

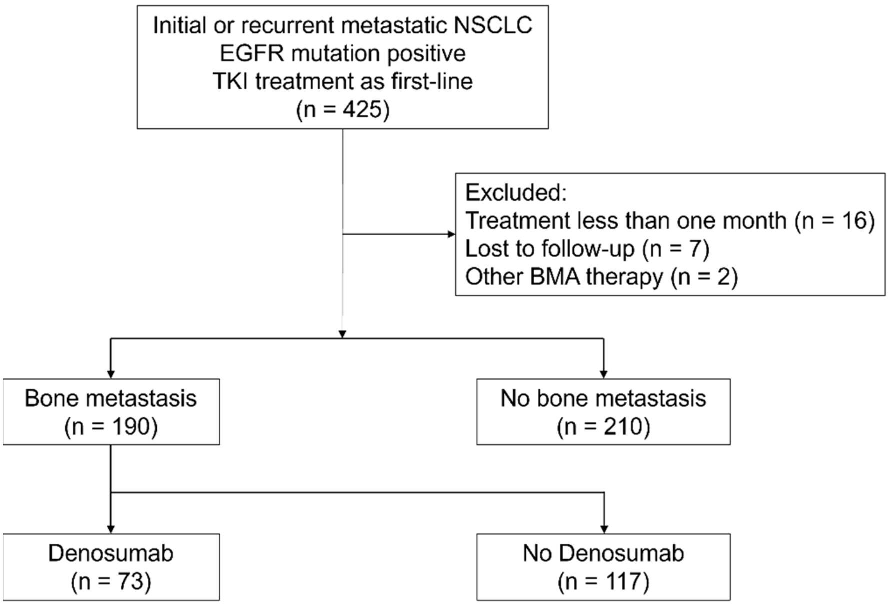

Figure S1. Flowchart of patient enrollment in this study. BMA, bone-modifying agent; *EGFR*, epidermal growth factor receptor; NSCLC, non-small cell lung cancer; TKI, tyrosine kinase inhibitor.

Table S1. Cox regression analysis of OS of all metastatic *EGFR*-mutated NSCLC patients.

Variable	Univariate analysis			Multivariate analysis		
	HR	95% CI	*p* value	HR	95% CI	*p* value
Age						
≥ 65	1.047	0.829–1.322	0.701	–	–	–
Sex						
Female	1.190	0.936–1.512	0.155	–	–	–
ECOG PS						
2–4	2.810	2.096–3.768	< 0.001	2.471	1.812–3.369	< 0.001
Smoking status						
Current/ex-smoker	0.995	0.744–1.331	0.975	–	–	–
Histology						
Adenocarcinoma	0.489	0.303– 0.789	0.003	0.566	0.343–0.935	0.026
Metastasis						
with lung/pleura/pericardia	1.049	0.807–1.364	0.718	–	–	–
with bone	1.595	1.262–2.016	< 0.001	1.372	1.069–1.760	0.013
with brain	1.197	0.938–1.526	0.149	–	–	–
with liver	1.638	1.178–2.277	0.003	1.322	0.909–1.924	0.144
with adrenal/renal	2.259	1.606–3.179	< 0.001	2.391	1.670–3.423	< 0.001
with abdominal LNs/spleen	1.388	0.913–2.111	0.125	–	–	–
EGFR mutation						
Exon 19 deletion	0.627	0.493	0.000	0.658	0.514–0.842	0.001
First-line *EGFR*-TKI						
Afatinib	0.817	0.644	0.096	–	–	–

CI, confidence interval; ECOG PS, Eastern Cooperative Oncology Group performance status; EGFR, epidermal growth factor receptor; HR, hazards ratio; LNs, lymph nodes; NSCLC, non-small cell lung cancer; OS, overall survival; TKI, tyrosine kinase inhibitor.
